# Supplementary material for: Conditional deletion of WT1 in the septum transversum mesenchyme causes congenital diaphragmatic hernia in mice
Source: eLife. 2016 Sep 19;5:e16009. doi: 10.7554/eLife.16009 (PMC5028188; doi:10.7554/eLife.16009)
Supplement: Figure 7—source data 1. — DOI: http://dx.doi.org/10.7554/eLife.16009.010 [file elife-16009-fig7-data1.docx]

**Measurements of the pleuropericardial opening** **used for figure 7D (microns).**

| Wild + RA | Mut + RA | Mut - RA |
| --- | --- | --- |
| \| 114,08 \| \| --- \| | 184,76 | 167,4 |
| 117,8 | 80,6 | 193,44 |
|  | 111,6 | 251,72 |
|  | 74,4 | 248 |
|  | 130,2 | 279 |
|  | 145,7 | 322,4 |
|  | 291,4 | 303,8 |
|  | 192,2 |  |
