# Supplementary material for: Conditional deletion of WT1 in the septum transversum mesenchyme causes congenital diaphragmatic hernia in mice
Source: eLife. 2016 Sep 19;5:e16009. doi: 10.7554/eLife.16009 (PMC5028188; doi:10.7554/eLife.16009)
Supplement: Supplementary file 1. — This experiment is described at the end of the first section of the results. DOI: http://dx.doi.org/10.7554/eLife.16009.011 [file elife-16009-supp1.docx]

**Image analysis data** of the volume taken up by the YFP+ cells in the right and left PHMP estimated by the total sum of the areas measured on serial sections. Data are square microns. This experiment is described at the end of the first section of the results.

|  | E11.5 | | E11.5 | | E12.5 | | E12.5 | |
| --- | --- | --- | --- | --- | --- | --- | --- | --- |
|  | L | R | L | R | L | R | L | R |
|  | 5518 | 31903 | 21823 | 10575 | 19467 | 62194 | 12169 | 28512 |
|  | 7613 | 32292 | 32577 | 5627 | 42622 | 46431 | 27602 | 59431 |
|  | 11658 | 22872 | 28329 | 6451 | 58270 | 60258 | 28077 | 28546 |
|  | 24266 | 18634 | 42102 | 66991 | 931 | 36034 | 24540 | 28717 |
|  | 21670 | 16055 | 25164 | 59420 | 46159 | 65302 | 12105 | 14760 |
|  | 62629 | 39394 | 22789 | 48591 | 50076 | 44157 | 15127 | 35641 |
|  |  |  | 17593 | 42691 | 42081 | 43155 | 63774 | 35355 |
|  |  |  | 36994 | 89407 |  | 40213 | 16788 | 54584 |
|  |  |  |  |  |  |  | 37121 | 42472 |
|  |  |  |  |  |  |  |  | 31228 |
|  |  |  |  |  |  |  |  | 6644 |
|  |  |  |  |  |  |  |  | 16061 |
| **Total** | 133354 | 161150 | 227371 | 329753 | 259606 | 397744 | 237303 | 381951 |
| **R/L ratio** | 1,21 | | 1,451 | | 1,53 | | 1,61 | |
